# VarStack: a web tool for data retrieval to interpret somatic variants in cancer

**DOI:** 10.1093/database/baaa092

**Published:** 2020-11-28

**Authors:** Morgan Howard, Bruce Kane, Mary Lepry, Paul Stey, Ashok Ragavendran, Ece D Gamsiz Uzun

**Affiliations:** Rhode Island Hospital Department of Pathology, Providence, RI 02903, USA; Computing and Information Services, Brown University, Providence, RI 02912, USA; Computing and Information Services, Brown University, Providence, RI 02912, USA; Brown University, Providence, RI, 02906, USA; Brown University, Providence, RI, 02906, USA; Center for Computational Biology of Human Disease, Brown University, Providence, RI 02912, USA; Rhode Island Hospital Department of Pathology, Providence, RI 02903, USA; Department of Pathology and Laboratory Medicine, Brown University Alpert Medical School, Providence, RI 02903, USA; Center for Computational Molecular Biology, Brown University, Providence, RI 02906, USA

## Abstract

Advances in tumor genome sequencing created an urgent need for bioinformatics tools to support the interpretation of the clinical significance of the variants detected. VarStack is a web tool which is a base to retrieve somatic variant data relating to cancer from existing databases. VarStack incorporates data from several publicly available databases and presents them with an easy-to-navigate user interface. It currently supports data from the Catalogue of Somatic Mutations in Cancer, gnomAD, cBioPortal, ClinVar, OncoKB, CiViC and UCSC Genome Browser. It retrieves the data from these databases and returns them back to the user in a fraction of the time it would take to manually navigate each site independently. Users submit a variant with a gene symbol, peptide change and coding sequence change. They may select a variety of tumor-specific studies in cBioPortal to search through in addition to their original query. The results from the databases are presented in tabs. Users can export the results as an Excel file. VarStack also has the batch search feature in which the user can submit a list of variants and download an Excel file with the data from the databases. With the batch search and data download options, users can easily incorporate VarStack into their workflow or tools. VarStack saves time by providing somatic variant information to the user from multiple databases in an easy-to-export and interpretable format. VarStack is freely available under https://varstack.brown.edu.

## Introduction

Advances in sequencing technologies and bioinformatics have improved precision medicine approaches by identifying tumor-specific variants. As a result, tumor variant data are large and growing. Interpretation of the variants detected is a crucial step for clinical decision-making. However, as there are multiple databases available providing information on the variants, interpretation of the variants could be time-consuming. Most cancer institutions or hospitals have in-house tools to retrieve variant data. Those tools needed to have most up-to-date information from the databases. An easy-to-interpret and user-friendly tool with up-to-date information would be beneficial for rapid clinical decision-making.

Catalogue of Somatic Mutations in Cancer (COSMIC) (1, 2), gnomAD (3), cBioPortal (4, 5), ClinVar (6), OncoKB (7), CiViC (8) and UCSC Genome Browser (9) are among the existing databases used by physicians and scientists for variant interpretation. COSMIC is the database of somatic mutations in cancer. It provides manually curated information on somatic mutations, fusions and mutations in cell lines (1, 2). gnomAD includes 71 702 genome sequencing data from unrelated subjects (3). cBioPortal is the database for nonsynonymous mutations, copy-number variations, mRNA and miRNA expression data from several genomic studies (4, 5). ClinVar provides information on variants and phenotypes (6). OncoKB is a knowledge-based database of variants, their effects and treatment options (7). CiViC is another database of variant information which allows users to provide their expert input (8). UCSC Genome Browser is a genome visualization tool with variant information (9). These databases facilitate specific variant information. For variant interpretation, access to one or more databases is needed, and reviewing multiple resources might be time-consuming. We developed VarStack 1.0 in order to provide somatic variant information from the multiple databases on a fast and easy-to-interpret platform with UCSC Genome Browser visualization.

## VarStack

VarStack is a web tool which retrieves up-to-date variant information from COSMIC, gnomAD, cBioPortal, ClinVar, OncoKB as well as UCSC Genome Browser and displays the output in separate tabs. It takes the gene name, amino acid and nucleotide change of a variant as an input. The COSMIC tab provides COSMIC ID, FATHMM prediction and coordinates in hg38 and Ensembl ID (1, 2). The ClinVar tab includes the type of variant, clinical significance, dbSNP ID as well as a link to dbSNP for the specific variant, phenotype list and coordinates in hg19 and hg38 (6, 10). VarStack also provides minor allele frequency information from gnomAD (3). As an option, the user selects specific studies from the cBioPortal list according to the tumor type (4). If this option is used, a graph with the frequency of the variant in the specific tumor type as well as a list of samples from the studies selected is provided under cBioPortal tab. The genomic location of the variant can be visualized on UCSC Genome Browser tab without navigating to the website (9). The user can review the data on the tabs or export it in an Excel file. OncoKB tab in the downloadable Excel file is split into three different tabs: the first contains the actionable variant information, the second contains the curated gene information and the last tab displays all of the annotated variant information.

VarStack also has a batch search feature which provides the data for a list of variants as a downloadable Excel file. The user enters the list of variants in a search box, and the web tool returns an Excel file with the data in separate tabs for ClinVar, COSMIC, gnomAD and OncoKB. With the data export and batch search options, VarStack could be easily incorporated into existing tools or workflows.

## System description

**Figure 1.**
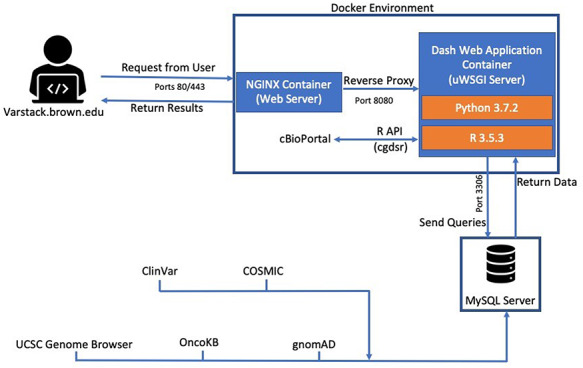
VarStack uses NGINX, R and Python to retrieve information from COSMIC, ClinVar, gnomAD, cBioPortal and OncoKB. An iframe of the UCSC Genome Browser is used to view the genomic location of the variant.

VarStack is comprised of several different components including Dash (Python framework), Python (v.3.7.2), MySQL and R (v.3.5.3). Dash uses Flask, React.js and Plotly.js to provide a framework for designing web applications. It was chosen to manage the user interface and connect to a MySQL database that stores the up-to-date data from COSMIC (1, 2), ClinVar (6), gnomAD (3) and OncoKB (7). The genomic location of the variant can be viewed through an iframe of the UCSC Genome Browser using GRCh38 coordinates retrieved from the COSMIC data table (9). R package cgdsr was used to retrieve data from cBioPortal studies (4) (Figure 1). cgdsr is an application programming interface (API) that accesses the Cancer Genomics Data Server.

## Discussion

The data retrieved and provided by VarStack could be obtained by manually navigating COSMIC, ClinVar, gnomAD, cBioPortal, OncoKB and UCSC Genome Browser. However, this would be time-consuming because it involved retrieving information for multiple variants. VarStack outputs the data in a user-friendly interface which is easy to navigate and download on one platform instead of navigating through separate websites. Data download option provides an Excel file containing the data provided in the user interface, and the user does not have to save the data separately from those databases. This file provides information similar to how it is displayed on VarStack in tabs with ClinVar, COSMIC, gnomAD, cBioPortal and OncoKB. The batch search feature provides data from ClinVar, COSMIC, gnomAD and OncoKB for multiple variants as a downloadable Excel file. Downloadable result file and batch search options make VarStack very compatible to be implemented for existing workflows or tools.

VarStack provides up-to-date information as it includes the recent updates in the databases, but does not include expert input. The databases included in VarStack are very well curated by experts. The existing knowledge-based databases, such as CIViC, provide expert input in addition to the information from specific databases. VarStack provides up-to-date information from commonly navigated databases for somatic variant interpretation in cancer.

## Limitations and future development

There are some limitations with the methods in this version of VarStack that we are aware of. The current release of VarStack provides somatic variant data from COSMIC, gnomAD, cBioPortal and OncoKB as well as UCSC Genome Browser visualization. We plan to extend the resource options to other databases such as CiViC, MyCancerGenome and ClinGen. The batch search does not contain the cBioPortal information as it requires specific tumor types. This feature will be implemented in the next versions. In addition, we plan to provide a transcript-based search option in the next release. Also, API access to the aggregated data will be available for the users in the future releases.

## Conclusions

VarStack is designed to be useful for the scientists and physicians who interpret somatic variants in multiple samples. Navigating multiple databases for several samples could be time-consuming. Also, certain databases require bioinformatics or programming knowledge. The easy-to-navigate user interface of VarStack does not require any bioinformatics or programming knowledge and saves time for the users. The tab-separated user interface is a practical solution in terms of providing information from multiple databases on one base with a downloadable file and batch search features.
